# Supplementary material for: Whole genome sequencing-based identification of human tuberculosis caused by animal-lineage Mycobacterium orygis
Source: J Clin Microbiol. 2023 Oct 25;61(11):e00260-23. doi: 10.1128/jcm.00260-23 (PMC10662373; doi:10.1128/jcm.00260-23)
Supplement: Data S3 — Custom in-house-developed R-scripts for sequence extraction and SNV identification from WGS sequences. [file jcm.00260-23-s0001.docx]

**Supplementary Data S3 Custom in-house-developed R-scripts for sequences extraction and SNVs identification from WGS sequences**

#Generic Profiler: Blast wild type genes vs. SampleNo_contigs.fasta

#R Studio scripting on local version of R Studio

#2022-05-15

#Walter Demczuk (Modified by Michelle Wuzinski and Md Rashedul Islam)

# MOR loci ------------------------------------------------------------------------------------Get User Input

#----------------------------------------------------------------------------------------------

Org_id <- "MOR" #MOR, MAF

Test_id <- "MASTER" #MASTER

SampleNo <- "list" #"/Location_Working_Directory/list.csv"

LocusID <-"Loci_Name" #in working directory "./temp/loci.csv"

Variable <- NA

#--------------------------------------------------------------------------------------------------------

switch(Org_id,

MOR={

ContigsDir <- "D:\\Genomes(Location_Contigs_Directory)\\"

switch(Test_id,

MASTER={setwd("D:\\Location_Working_Directory\\")}

)

},

{

Switch_entry <- "NOTHING"

}

)

#------------------------------------------------------------------------------------------------------------

# Libraries####

library(dplyr)

library(stringr)

library(Biostrings)

#------------------------------------------------------------------------------------------------------####

# Code starts here ####

unlink("./temp/output_dna.fasta") #this deletes the file!

unlink("./temp/output_dna_notfound.fasta")

# unlink("./temp/output_aa.fasta") #this deletes the file!

# unlink("./temp/output_aa_notfound.fasta") #this deletes the file!

if(SampleNo == "list")

{

SampleList.df <- read.csv("list.csv", header = TRUE, sep = ",", stringsAsFactors = FALSE)

}else

{

SampleList.df <- data.frame(SampleNo, Variable)

}

Size.df <- dim(SampleList.df)

NumSamples <- Size.df[1]

if(LocusID == "list")

{

LocusList.df <- read.csv("./temp/loci.csv", header = TRUE, sep = ",", stringsAsFactors = FALSE)

#LocusList.df <- read.csv("./temp/loci_short.csv", header = TRUE, sep = ",", stringsAsFactors = FALSE) #for testing

}else

{

LocusList.df <- data.frame(LocusID)

}

SizeList <- dim(LocusList.df)

NumLoci <- SizeList[1]

#wwwwwwwwwww

for( z in 1L:NumLoci) #Format Blast databases

{

locus <- as.character(LocusList.df[z,])

#Index Blast Lookup files-----------------------------------------------------

LocusLkupDNA <- paste("./allele_lkup_dna/", locus, ".fasta", sep = "")

if(file.exists(LocusLkupDNA))

{

LocusLkupDNApresent = TRUE

BlastFormatCommand <- paste("makeblastdb -in ", LocusLkupDNA, " -dbtype nucl", sep = "")

try(system(BlastFormatCommand))

}

}

#wwwwwwwwww

for (m in 1L:NumSamples) #<<<<<<<<<<<<<<<<<<<<<<<<<<<<<<<<<<<<<<<<<<<<<<<<<<<<<<<<<<<<<<<<<<<<<<<<<<<<<<<

{

CurrSampleNo <- as.character(SampleList.df[m, "SampleNo"])

CurrSampleVar <-as.character(SampleList.df[m, "Variable"])

CurrSample.df <- filter(SampleList.df, SampleNo == CurrSampleNo)

SampleProfile <- ""

for(p in 1L:NumLoci) #xxxxxxxxxxxxxxxxxxxxxxxxxxxxxxxxxxxxxxxxxxxxxxxxxxxxxxxxxxxxxxxxxxxxx

{

locus <- as.character(LocusList.df[p,])

#Index Blast Lookup files-----------------------------------------------------

LocusLkupDNA <- paste("./allele_lkup_dna/", locus, ".fasta", sep = "")

#D:\Location_Working_Directory\allele_lkup_dna

if(file.exists(LocusLkupDNA))

{

LocusLkupDNApresent = TRUE

#BlastFormatCommand <- paste("makeblastdb -in ", LocusLkupDNA, " -dbtype nucl", sep = "")

#try(system(BlastFormatCommand))

}else

{LocusLkupDNApresent = FALSE

}

AlleleLine <- ""

AlleleInfo <- NA

AlleleInfo[1] <- NA

AlleleInfo[2] <- NA

AlleleInfo[3] <- NA

AlleleInfo[4] <- NA

IdentLine <- ""

LocusFile <- paste("./wildgenes/", locus, ".fasta", sep = "")

if(!file.exists(LocusFile))

{

stop("Wild Type locus file not found!")

}

# .....................................................................BLAST and parse blastout.txt

QueryFile <- paste(ContigsDir, CurrSampleNo, ".fasta", sep = "")

if (!file.copy(QueryFile, "./temp/queryfile.fasta", overwrite = T))

{

stop("Sample number not found in contigs directory!")

} else

{

try(shell("makeblastdb -in ./temp/queryfile.fasta -dbtype nucl", intern = TRUE))

#BlastCommand <- paste("blastall -i", LocusFile, "-d ./temp/queryfile.fasta -p blastn -o ./temp/blastout.txt -v 0 -b 1 -e 10e-85 -F F")

BlastCommand <- paste("blastn -query", LocusFile, "-db ./temp/queryfile.fasta -out ./temp/blastout.txt -num_descriptions 0 -num_alignments 1 -evalue 10e-50")

try(system(BlastCommand))

FileName <- "./temp/blastout.txt"

con <- file(FileName, open="r")

linn <- readLines(con)

close(con)

#check if gene was found in BLAST

BlastResult <- NA

for (i in 1:length(linn))

{

if (str_detect(linn[i], "No hits found"))

{

BlastResult <- "NEG"

AlleleInfo[1] <- "NEG"

AlleleInfo[2] <- ""

AlleleInfo[3] <- ""

AlleleInfo[4] <- ""

break()

} else

{

BlastResult <- "POS"

AlleleInfo[1] <- "POS"

AlleleInfo[2] <- ""

AlleleInfo[3] <- ""

AlleleInfo[4] <- ""

}

}

if (BlastResult == "POS")

{

DNASeqLine_str = ""

WTDNASeqLine_str = ""

for (i in 1:length(linn))

{

if (str_detect(linn[i], "Identities"))

{

IdLine <- unlist(linn[i])

IdLine <- substr(IdLine, 15, 50)

}

if (str_detect(linn[i], "Query "))

{

QueryLine <- unlist(strsplit(linn[i], " "))

QueryLine <- QueryLine[QueryLine != ""]

WTDNASeqLine_str <- paste(WTDNASeqLine_str, QueryLine[3], sep = "")

}

if (str_detect(linn[i], "Sbjct "))

{

SbjctLine <- unlist(strsplit(linn[i], " "))

SbjctLine <- SbjctLine[SbjctLine != ""]

DNASeqLine_str <- paste(DNASeqLine_str, SbjctLine[3], sep = "")

}

}

WTDNASeqLine <- DNAString(WTDNASeqLine_str)

WTDNASeqLine_NoDash_str <- str_replace_all(WTDNASeqLine_str, "-", "")

WTDNASeqLine_NoDash <- DNAString(WTDNASeqLine_NoDash_str)

if (SampleNo != "list")

{

cat("\n\n>", locus , "(Wildtype)\n", WTDNASeqLine_NoDash_str, sep ="")

}

DNASeqLine <- DNAString(DNASeqLine_str)

DNASeqLine_NoDash_str <- str_replace_all(DNASeqLine_str, "-", "")

DNASeqLine_NoDash <- DNAString(DNASeqLine_NoDash_str)

if (SampleNo != "list")

{

cat("\n\n>", CurrSampleNo, "_", locus , "\n", DNASeqLine_NoDash_str, sep ="")

}

#-------------------------------------------------------------------------------make Protein sequence

WTAASeqLine <- translate(WTDNASeqLine_NoDash)

WTAASeqLine_str <- toString(WTAASeqLine)

if (SampleNo != "list")

{

cat("\n\n>", locus , "(Wildtype)\n", WTAASeqLine_str, sep ="")

}

AASeqLine <- translate(DNASeqLine_NoDash)

AASeqLine_str <- toString(AASeqLine)

# AASeqLine_str <- str_replace_all(AASeqLine_str, "[*]", "")

if (SampleNo != "list")

{

cat("\n\n>", CurrSampleNo, "_", locus , "\n", AASeqLine_str, sep ="")

}

#-----------------------------------make sure AASeqLine, WTAASeqLine are listed in correct order!!!!!!!!!!!!

globalAlign_AA <- pairwiseAlignment(AASeqLine, WTAASeqLine, substitutionMatrix = "BLOSUM50", gapOpening = -2,

gapExtension = -8, scoreOnly = FALSE)

WTAASeqLine_aln <- subject(globalAlign_AA)

WTAASeqLine_aln_str <- toString(WTAASeqLine_aln)

#cat("\n\n>", locus, "_alignment\n", WTAASeqLine_aln_str, sep = "")

AASeqLine_aln <- pattern(globalAlign_AA)

AASeqLine_aln_str <- toString(AASeqLine_aln)

#cat("\n\n>", SampleNo, "_", locus , "_alignment\n", AASeqLine_aln_str, sep = "")

DNASeqLine_aln <- ""

for (j in 1:str_length(WTDNASeqLine_str))

{

if (str_sub(WTDNASeqLine_str, j, j) == str_sub(DNASeqLine_str, j, j))

{

DNASeqLine_aln <- paste(DNASeqLine_aln, ".", sep = "")

} else

{

DNASeqLine_aln <- paste(DNASeqLine_aln, str_sub(DNASeqLine_str, j, j), sep = "")

}

}

if (SampleNo != "list")

{

cat("\n\nDNA Alignment:\n", DNASeqLine_aln, sep = "")

}

#------------------------------------------------------------------------------mutations

AASeqLine_aln_disp <- ""

mutations <- ""

for (k in 1:str_length(WTAASeqLine_aln_str))

{

if (str_sub(WTAASeqLine_aln_str, k, k) == str_sub(AASeqLine_aln_str, k, k))

{

AASeqLine_aln_disp <- paste(AASeqLine_aln_disp, ".", sep = "")

} else

{

AASeqLine_aln_disp <- paste(AASeqLine_aln_disp, str_sub(AASeqLine_str, k, k), sep = "")

mutations <- paste(mutations, str_sub(WTAASeqLine_aln_str, k, k), k, str_sub(AASeqLine_str, k, k), " ", sep = "")

}

}

if (SampleNo != "list")

{

cat("\n\nProtein Alignment:\n", AASeqLine_aln_disp, "\n", sep = "")

cat("\n", mutations, sep = "")

}

#----------------------------------------------------------------------------------Lookup Alleles DNA

# write DNASeqLine_NoDash_str to a file,

# BLAST vs. lookup table,

# parse out the allele numbers

sink("./temp/querygene.fasta", split=FALSE, append = FALSE)

cat(">", CurrSampleNo, "_", locus , "\n", DNASeqLine_NoDash_str, sep ="")

sink()

#unlink("./temp/querygene.fasta") #this deletes the file!

#file.show("./temp/querygene.fasta")

ExactMatchFound <- FALSE

if(LocusLkupDNApresent)

{

#BLAST lookup table

BlastCommand2 <- paste("blastn -query ./temp/queryfile.fasta ", "-db ", LocusLkupDNA, "-out ./temp/blastout2.txt -num_descriptions 0 -num_alignments 1 -evalue 10e-85")

try(system(BlastCommand2))

FileName2 <- "./temp/blastout2.txt"

con <- file(FileName2, open="r")

linn <- readLines(con)

close(con)

#parse blastout2

for (i in 1:length(linn))

{

if (str_detect(linn[i], "Identities"))

{

IdentLine <- unlist(linn[i])

IdentLine <- substr(IdentLine, 15, 50)

if (str_detect(IdentLine, "100%"))

{

ExactMatchFound <- TRUE

}

}

if (str_detect(linn[i], ">"))

{

AlleleLine <- unlist(linn[i])

AlleleLine <- substr(AlleleLine, 2, 50)

AlleleParts <- strsplit(AlleleLine, "_")

AlleleParts2 <- unlist(AlleleParts)

}

}

if(ExactMatchFound) #if only not found in fasta make files here.

#AlleleParts2[1] holds POS/NEG; [2] allele number; [3] mutations or WT; [4] extra info

{

AlleleInfo[2] <- AlleleParts2[2]

AlleleInfo[3] <- AlleleParts2[3]

AlleleInfo[4] <- AlleleParts2[4]

}else

{

AlleleInfo[2] <- "NF"

AlleleInfo[3] <- ""

AlleleInfo[4] <- ""

}

}#close bracket for DNA lookup file exists check

#-------------------------------------------write a fasta file of all sequences output_dna.fasta and output_aa.fasta

sink("./temp/output_dna.fasta", split=FALSE, append = TRUE)

cat(">", locus, "_", CurrSampleNo, "_", locus, "_", AlleleInfo[3], "_", CurrSampleVar, "\n", DNASeqLine_NoDash_str, "\n", sep ="")

sink()

if (AlleleInfo[2] == "NF")

{

sink("./temp/output_dna_notfound.fasta", split=FALSE, append = TRUE)

cat(">", locus, "_", CurrSampleNo, "_", locus, AlleleInfo[3], "_", CurrSampleVar, "\n", DNASeqLine_NoDash_str, "\n", sep ="")

sink()

}

sink("./temp/output_aa.fasta", split=FALSE, append = TRUE)

cat(">", locus, "_", CurrSampleNo, "_", CurrSampleVar, "\n", AASeqLine_str, "\n", sep ="")

sink()

}else #close bracket for BLAST positive

{

# cat(CurrSampleNo, "\t", locus, "\tBLAST Negative\n", sep = "")

}

col1_name <- paste(locus, "_result", sep = "")

col2_name <- paste(locus, "_allele", sep = "")

col3_name <- paste(locus, "_mutations", sep = "")

col4_name <- paste(locus, "_comments", sep = "")

headers <- list(col1_name, col2_name, col3_name, col4_name)

if(p==1)

{

OutputLocus.df <- data.frame(AlleleInfo[1], AlleleInfo[2], AlleleInfo[3], AlleleInfo[4], stringsAsFactors = FALSE)

names(OutputLocus.df) <- headers

}else

{

OutputLocus2.df <- data.frame(AlleleInfo[1], AlleleInfo[2], AlleleInfo[3], AlleleInfo[4], stringsAsFactors = FALSE)

names(OutputLocus2.df) <- headers

OutputLocus.df <- cbind(OutputLocus.df, OutputLocus2.df)

}

} #close bracket for contig file found.

ProfileEntry <- ""

if (AlleleInfo[1] == "POS")

{

if (LocusLkupDNApresent)

{

if (AlleleInfo[3] == "" | is.na(AlleleInfo[3]))

{

ProfileEntry<-locus

}else if (AlleleInfo[3]=="WT" )

{ProfileEntry<-""}else

{ProfileEntry <- paste(locus, AlleleInfo[3], sep = " ")}

}else

{ProfileEntry<-locus}

if (SampleProfile == "")

{

SampleProfile <- ProfileEntry

}else

{

if (ProfileEntry!=""){ProfileEntry<-paste("-", ProfileEntry, sep = "")}

SampleProfile <- paste(SampleProfile, ProfileEntry, sep = "")

}

}

cat(CurrSampleNo, locus, AlleleInfo[1], AlleleInfo[2], AlleleInfo[3], AlleleInfo[4], "\n", sep = "\t")

} #end of locus list loop xxxxxxxxxxxxxxxxxxxxxxxxxxxxxxxxxxxxxxxxxxxxxxxxxxxxxxxxxxxxxxxxxxxxxxxxxxxxxxxx

cat("\n", CurrSampleNo, " Molecular Profile: ", SampleProfile, "\n\n", sep = "")

SampleProfile.df <- data.frame(SampleProfile)

OutputLocus.df <- cbind(CurrSample.df, OutputLocus.df, SampleProfile.df)

#OutputLocus.df <- cbind(CurrSample.df, OutputLocus.df, SampleProfile.df, AMR.df)

if(m==1) #if first sample make one row profile table, otherwise add new row to table

{

OutputProfile.df <- data.frame(OutputLocus.df)

}else

{

OutputProfile.df <- rbind(OutputProfile.df, OutputLocus.df)

}

} #close brack for sample list loop<<<<<<<<<<<<<<<<<<<<<<<<<<<<<<<<<<<<<<<<<<<<<<<<<<<<<<<<<<<<<<<<<<<<<<<<<

View(OutputProfile.df)

write.csv(OutputProfile.df, "./temp/output_profile.csv", row.names = F)
